# The relationship between toxic leadership and organizational performance: the mediating effect of nurses’ silence

**DOI:** 10.1186/s12912-022-01167-8

**Published:** 2023-01-04

**Authors:** Sally Mohammed Farghaly Abdelaliem, Mennat Allah G. Abou Zeid

**Affiliations:** 1 Associate Professor, Departement of Nursing Management and Education, College of Nursing, Princess Nourah bint Abdulrahman University, Riyadh, Saudi Arabia; 2 Associate Professor at Nursing Administration Departement, Faculty of Nursing, Alexandria University, Alexandria, Egypt; 3 Lecturer, Nursing Administration Department, Faculty of Nursing Ain Shams University, Cairo, Egypt

**Keywords:** Toxic leadership, Organisational performance, Nurses’ silence, University Hospital

## Abstract

**Aims:**

To assess toxic leadership and organizational performance among nurses of a University Hospital, and explore the mediating effect of nurses ‘silence.

**Background:**

Toxic Leadership behaviours are hurtful to feelings, leading to emotive fatigue and nurses silence within the legislative framework. In fact, it is very harmful to all organizations. However, little emphasis has been paid to research on the mediating mechanism and border factors of their connection.

**Methods:**

A cross-sectional design was applied. Data was collected from 750 nurses over the course of three months. The researchers employed structured equation modeling [SEM] to investigate the role of nurses’ silence in mediating the association between toxic leadership and organizational performance.

**Results:**

The toxic leadership level and Organisational performance level were both high. In addition to, the nurses’ silence level was moderate. The SEM revealed that toxic Leadership accounted for the prediction of 65% of the variance of nurses’ silence and 87% of the variance of organizational performance. Also, nurses silence as a mediating factor accounted for 73% of the variance of organizational performance.

**Conclusions:**

This study emphasized on the importance of creating a work environment that encourages and promotes open communication, as well as eliminating toxic leadership behaviours from the organizational culture among nurses as it effects on the organizational performance.

## Introduction

Every corporation, particularly health institutions, requires people with exceptional business performance to survive and prosper, adapt to changing external conditions, and maintain a competitive advantage. Leaders can try to improve employees' job performance by creating a positive work environment and fostering an optimistic organizational climate through arrangements that encourage individuals to be optimistic [1]. Effective leadership practices based on the values of respect, trust, and open communication are critical not only in providing high-quality care, but also in creating a quality work environment where nurses are respected and valued, which helps to keep them motivated, satisfied, and committed to the organization [2]. Ineffective leadership practices are becoming a growing concern in the healthcare and nursing fields, with negative implications for nurse job outcomes and performance. As nurses are the largest professional group in healthcare, it is critical to investigate the impact of their silence on the relationship between toxic leadership behaviours and organizational performance [3, 4].

## Background

### Toxic leadership

Orukwowu, (2022) [5] defined leadership as the “process by which an individual influences a group of in which the leader affects his or her followers while also being affected by them, making it a transactional event. Furthermore, leadership entails “influencing followers” and is defined by the leader's ability to affect and influence their followers effectively. Toxic leadership, a type of ineffective leadership, is becoming more common in management literature and has piqued the interest of many researchers in recent years [6, 7]. Toxic leadership is a negative leadership style in which a leader engages in systematic and destructive behaviours that harms individuals and organizations directly or indirectly [8]. Hoffman and Sergio (2020) [9] defined toxic leadership as disregarding employees' well-being and participating in actions and activities that demean, belittle, and discourage employees. They also micromanage, are rude, do not listen, and are threatening to their employees. Furthermore, they abuse their power, which lowers employee job satisfaction and morale. A few variables known to cause toxicity in leaders have been identified in the literature, including corporate culture, instability, perceived threat, and successful institutions. Toxic leaders thrive in organizations that promote high performance but lack mechanisms to assess how these goals and objectives are met [10, 11]. Furthermore, some toxic workplace leadership behaviours include: criticizing subordinates for flaws, demanding job expectations, lambasting employees' work skills, insulting, demeaning an individual's triumphs, and considering others' work [12]. Toxic leaders, according to researchers, are harmful to employee and organizational performance because of disparaging and self-serving behaviours aimed at achieving personal goals and benefits by exploiting or compromising the needs and desires of subordinates, teams, and organizations [13]. Furthermore, toxic leadership has an impact on both organizational and individual performance.

### Organizational performance

The organizational performance is determined by whether the specific leadership style is appropriate for the organization's specific situation [14]. Leaders' behaviours cause the emergence of positive behaviours and psychological conditions in employees [15], whereas toxic leadership is a threat to positive employee behaviour and performance [16]. Organizational performance is defined as “the indicator that assesses the organization’s effectiveness in achieving its objectives”. The efficiency and effectiveness with which the company achieves its goals can be used to evaluate its performance. It includes an organization’s current output or outcomes [17].

For nurses, there are two types of job performance: task performance and contextual performance [18]. The task performance assesses how well nurses carry out the activities and responsibilities outlined in the official job description [19]. Contextual performance, on the other hand, is individual effort that is not directly related to their primary task function, but is critical because it serves as a significant stimulus for task activities. Nursing performance in both forms contributes to the effectiveness of health care organizations [20]. Previous research has identified a few organizational elements that may improve the job motivation and performance of nurses. Empowerment, autonomy, engagement, supervision and management, nature of work, professional training and learning opportunities, supportive relationships and communication, contingent rewards, pay and financial benefits, promotion opportunities, equity and organizational justice, and working conditions were proposed by Baljoon et al., (2018) [21].

### Employee silence

Employee silence is a result of toxic leadership [22], as employees prefer to remain silent, especially when confronted with self-centred and self-serving toxic leaders [23–25]. Employee silence is defined as “any truthful declaration of an individual’s behavioural, cognitive, and/or affective appraisal of his or her organizational conditions withheld from others deemed capable of influencing change” [26]. According to reports, employee silence is a barrier to openness, effective decision making, innovation, the change process, and continuous improvement [27, 28]. Employees may become frustrated, dissatisfied with their jobs, and eventually leave [29, 30]. Furthermore, employee silence has a negative impact on organizational outcomes such as ineffective decision-making, a lack of innovation, learning, and change adoption [27]. According to the study, regulatory reasons, anxiety about destroying relationships with coworkers, fear of fines, fear of being isolated, lack of management support, and fear of being ignored are all factors that contribute to nurses’ silence [30–32]. Previous research has found that nurses with more nursing and employment experience, as well as those who are older, have higher levels of quietness [33]. Organizational elements that encourage nurses to remain silent include an unfair culture, a lack of psychological safety, a quiet climate, a hierarchical structure, a negative leadership style and lack of confidence in supervisors, and a hostile nursing work environment [32].

**Fig. 1:**
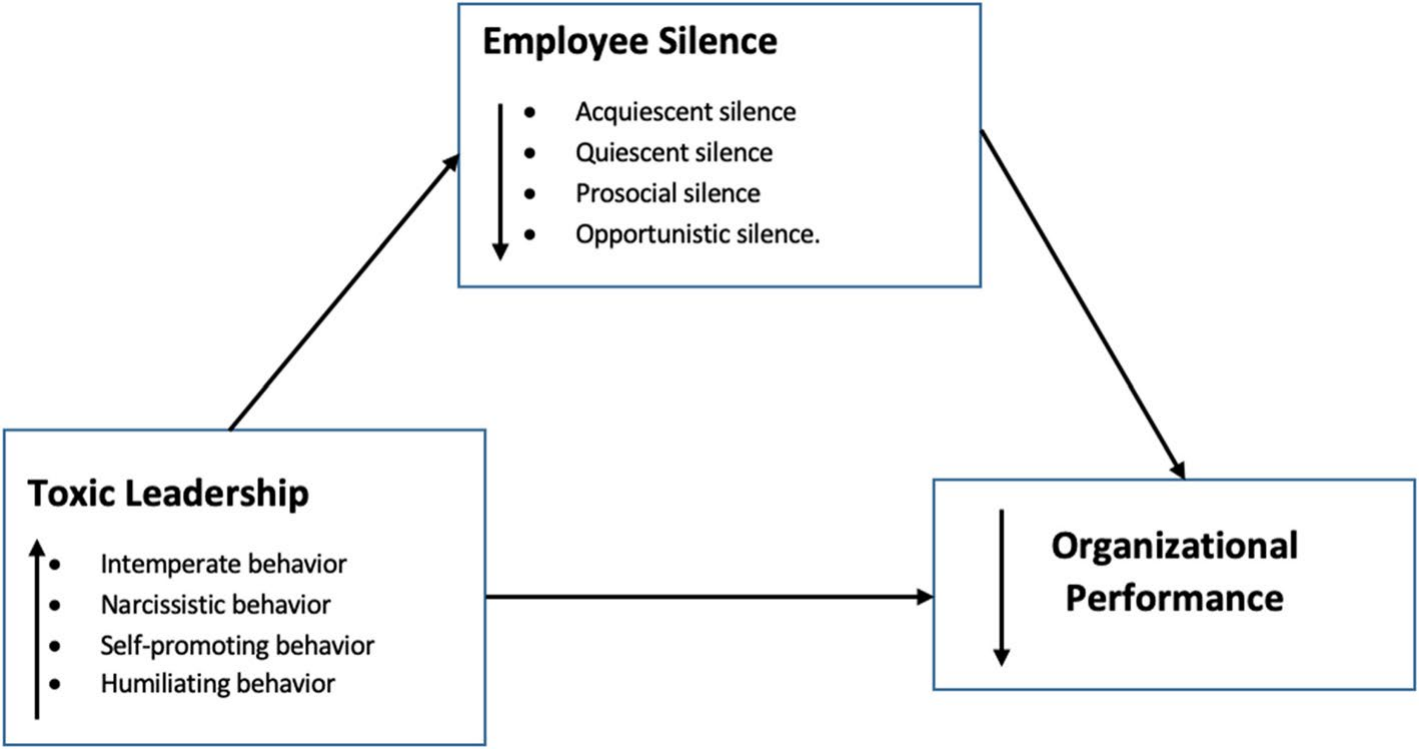
Reciprocal relationship between toxic leadership and organizational performance mediated by employee silence

The gap of knowledge and evidence on how toxic leadership and employee silence influence organizational performance in nursing professionals is very concerning. As a result, understanding these variables is critical when designing interventions or developing organizational policies to manage or prevent toxic behaviours, as well as to facilitate speaking-up behaviours and effective communication skills among nurses and their leaders. Furthermore, it is hoped that this research will untangle the tangled threads of toxic leadership and employee silence, and that there will be no more toxic leadership or employee silence among nursing professionals in the future. The expected reciprocal link between toxic leadership and organizational performance, as mediated by nurses’ silence, is depicted in Fig. 1. The current study sought to investigate the relationship between toxic leadership and organizational performance among nurses, as well as the moderating role of nurses’ silence. The study’s research questions were, “Is there a link between toxic leadership and organizational performance among nurses?” and “What influence does nurses’ silence have on toxic leadership and organizational performance?”.

## Methods

### Research design and setting

This is a cross-sectional, correlational, descriptive, quantitative study that was done at Alexandria Main University Hospital’s inpatient medical, surgical, and critical care units. The hospital had 1,825 beds, of which 952 were for medical treatment and its specialties, 773 were for surgical care and its specialties, and 100 were for critical care. Alexandria Main University Hospital is the city’s major teaching hospital. It had offered a comprehensive range of clinical acute treatments to the people of Alexandria and the surrounding governorates. Furthermore, it had provided chances for teaching and clinical training for medical and nursing students, as well as a venue for a range of scientific projects.

### Participants

The researchers employed a whole-population sampling strategy [purposive sampling technique]. The participants were chosen from a population of 750 nurses with at least six months of experience working in the previously defined units and who were available at the time of data collection. And the study’s subjects expressed an interest in participating in this examination. Nurses in training were not permitted to take part. Out of the 750 eligible nurses, 750 responded, yielding a 100% response rate.

### Study instruments

Gender, age, marital status, working unit, educational qualifications, years of nursing experience, years of experience in the present working unit, and working shift were among the demographic data collected. Toxic Leadership was assessed using the Toxic leadership scale [4]. This scale consisted of 30 items under four dimensions: intemperate (15 items; α = 0.868), narcissistic [9 items; α = 0.885], self-promoting (3 itemsα = 0.899), and humiliating behaviours (3 items; α = 0.877). Items on the intemperate behaviour subscale represent antagonistic actions or behaviours—verbal or nonverbal—repeated by nurse supervisors toward subordinates, indicating a lack of emotional intelligence. Items on the narcissistic behaviour subscale represented behaviours or acts that were largely motivated by personal goals and self-absorption. The self-promoting behaviour subscale included items that address activities or behaviours shown to improve one’s own personal or professional growth and advancement. Finally, the subscale of humiliating behaviour included items that represent activities that might disgrace or shame personnel. The participants rate each item on a 5-point scale (1 = not at all, 5 = frequently). The researchers computed the average score for each dimension as well as the overall scale score (α = 0.976) was the average of the four dimensions; higher scores indicate higher level of toxic leadership. The total score, which ranges from 20 to 150 points, can be regarded as essentially harmless (30–69 points), moderately harmful (70–110 points), or severely toxic (111–150 points). The concept validity, criteria validity, and internal consistency reliability of the scale were all determined to be adequate (0.975) [4].

To assess organizational performance, the researchers used the organizational performance questionnaire [34, 35]. This questionnaire is made up of eleven items that offer information regarding the organization’s communication, policies, development and change, and organizational performance appraisal. Participants rated each item on a 5-point scale (1 = never satisfied, 5 = extremely satisfied). The average score for the entire scale score was calculated by the researchers (α = 0.956). The mean scores were then determined, and they were classified as follows: mean scores < 3 = unsatisfied, and mean scores ≥ 3 = satisfied; higher scores implied more satisfaction with organizational performance. Furthermore, the nurses silence scale [36] was used to measure nurses silence (2013). This scale had 12 items divided into four categories: acquiescent (3 items; = 0.856), quiescent (3 items; = 0.785), prosocial (3 items; = 0.859), and opportunistic silence (3 items; = 0.977). Participants rated each item on a seven-point scale (1 = never, 7 = very frequently). The average score for each component was obtained by the researchers, and the overall scale score (α = 0.892) was the average of the four dimensions; higher scores implied a higher level of nurses’ silence.

### Validity and reliability

The three tools were adjusted, then translated into Arabic and back into English. The tools were then submitted to a panel of five experts (four Professors and one Lecturer from the Nursing Administration Department) who examined and assessed the content validity and offered feedback on the content, question types, and item clarity. Their comments were considered to ensure accuracy and to prevent possibly undermining the study. To examine the reliability of research tools, the internal consistency of items was measured using the Cronbach’s alpha coefficient test. At a statistical significance level of *p* ≤ 05., the three tools were determined to be reliable, with = 0.97 for tool one, 0.95 for tool two, and 0.89 for tool three. The pilot study was done on 10% of the staff nurses (*n* = 75) from the previously mentioned context to assess item clarity and practicality, identify potential hurdles and concerns during data collection, and test the time necessary to complete the tools. Some aspects need clarification from researchers, but did not necessitate change. Participants in the pilot study were not included in the study sample.

### Data collection

Data was gathered via survey questionnaires, which were distributed individually to nursing workers. Data was collected for three months, from November 1^st^, 2021 to January 30^th^, 2022. Due to their continuous presence at the hospital for training and instruction, nursing trainees were engaged to distribute and collect completed forms. Because they were commonly linked to particular healthcare professionals, they could quickly follow-up on distribution and collection. Participants received little presents in exchange for their participation. All participant questions were thoroughly answered and clarified.

### Ethical considerations

The Institutional Review Board of Faculty of Nursing, Ethics Committee at Alexandria University in Egypt (SN: 2022–9-82, IRB00013620) excused the study from ethical assessment. The subjects gave informed consent after being told about the goal of the study. Confidentiality and anonymity were ensured by assigning a code number to each questionnaire. Nurses were assured that their information would be kept strictly confidential and used only for research purposes. The ability to exit the study at any time has been ensured.

### Data analysis

SPSS version 23 was used to analyse the collected data. To quantify demographic and work-related characteristics, descriptive statistics (frequency, means, standard deviations, and percentages) were used, whereas inferential statistics such as the Student’s t test and analysis of variance (ANOVA) were used to compare toxic leadership, organizational performance, and nurses behaviour subscales between groups based on socio-demographic characteristics. To analyse the link between the variables in the study, the correlation coefficient was used. To predict the employee silence score and organizational performance in response to toxic leadership, a multiple regression analysis was undertaken. The variables included as independent variables in the multiple regression models were those that were statistically significant (*p* -value ≤ 0.05) in the correlational analysis, with a correlation coefficient of 100. Employee silence and statistically significant related socio-demographic factors were included to investigate their role as mediators in the relationship between toxic leadership and organizational performance. The mediating effect of employee silence was investigated using structural equation modeling.

## Results

After following up with all participants, the response rate was 100%. The majority of participants (78.3%) were females, with more than three-fifths of them being above the age of 30. A little less than one-fifth were working in medical and critical care units. The majority of the nurse respondents (*n* = 522; 69.6%) had a bachelor’s degree in nursing. Table 1 showed the socio-demographic characteristics of the respondents.

**Table 1:** Socio-demographic characteristics of participants (*n* = 750)

Socio-demographic characteristics	No	%
**Gender**		
Male	163	21.7
Female	587	78.3
**Age (years)**		
< 35	262	34.9
≥ 35	488	65.1
**Marital Status**		
Single	195	26.0
Married	392	52.3
Divorced	33	4.4
Widowed	130	17.3
**Working Unit**		
Medical unit	294	39.2
Surgical unit	163	21.7
ICU	293	39.1
**Educational Qualifications**		
Diploma in Nursing	32	4.3
Bachelor of Nursing Science	522	69.6
Master of Nursing Science	196	26.1
**Years of Experience in Nursing**		
< 5	326	43.5
5–10	424	56.5
**Years of Experience in the Current Working Unit**		
< 5	490	65.3
5–10	260	34.7
**Working Shift**		
Fixed morning	164	21.9
Rotating morning and evening	130	17.3
Rotating morning, evening and night	456	60.8

According to Table 2, the overall toxic leadership level was high (78.3%), with a mean score of 3.91 ± 0.51. In terms of individual subscales, the “Self-promoting behaviour” subscale had the greatest mean score (3.97 ± 0.19), while the “Humiliating behaviour” subscale had the lowest (3.56 ± 0.55). Furthermore, the total performance level of the organization was high (69.6), with a mean score of (4.22 ± 0.62). The “Humiliating behaviour” subscale had the highest proportion of respondents (47.9%) who rated it as “moderate-level”. Table 2 also demonstrated that the general degree of nurses’ silence was largely moderate (56.5%), with a mean score of 4.90 ± 0.72. In terms of individual subscales, the “Acquiescent silence” subscale had the highest mean score (5.52 ± 0.63).

**Table 2:** Mean Score and Level of Toxic Leadership, Organizational Performance and Nurse Silence (*n* = 750)

**Study Variables**	**Mean score**	Low (< **33.3%)**		Moderate (33.3%- < **66.67%)**		High (≥ **66.67%)**	
	**Mean ± SD**	**No**	**%**	**No**	**%**	**No**	**%**
**Toxic leadership**							
Intemperate behavior	4.01 ± 0.59	0	0.0	163	21.7	587	78.3
Narcissistic behavior	3.85 ± 0.61	0	0.0	326	43.5	424	56.5
Self-promoting behavior	3.97 ± 0.19	0	0.0	0	0.0	750	100.0
Humiliating behavior	3.56 ± 0.55	0	0.0	359	47.9	391	52.1
**Overall Toxic leadership**	**3.91 ± 0.51**	**0**	**0.0**	**163**	**21.7**	**587**	**78.3**
**Organizational performance**	**4.22 ± 0.62**	**0**	**0.0**	**228**	**30.4**	**522**	**69.6**
**Employee Silence **							
Acquiescent silence	5.52 ± 0.63	0	0.0	98	13.1	652	86.9
Quiescent silence	4.22 ± 1.06	130	17.3	294	39.2	326	43.5
Prosocial silence	4.87 ± 0.95	0	0.0	130	17.3	620	82.7
Opportunistic silence	5.07 ± 1.0	0	0.0	130	17.3	620	82.7
**Overall Employee Silence**	**4.90 ± 0.72**	**0**	**0.0**	**424**	**56.5**	**326**	**43.5**

**Fig. 2:**
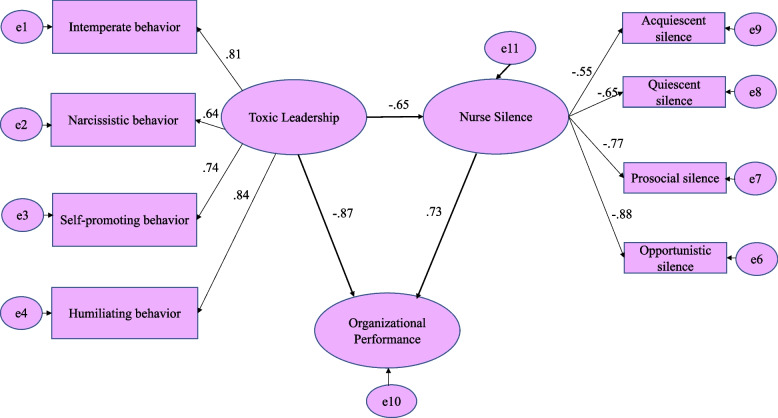
Standardized coefficients for path analysis of direct and indirect effect of toxic leadership on organizational performance mediated by nurses’ resilience

Regarding the correlation analysis in Table 3, a strong, negative, and significant correlation were noted not only between organizational performance and overall toxic leadership (*R*-value = -0.666 and *p*-value = 0.001), but also with all subscales of toxic leadership which were intemperate behaviour (*R*-value = -0.666 and *p*-value = 0.001), narcissistic behaviour (*R*-value = -0.608 and *p*-value = 0.001), self-promoting behaviour (*R*-value = -0.582 and *p*-value = 0.001), and humiliating behaviour (*R*-value = -0.483 and *p*-value = 0.001). In addition, a strong, negative and significant correlation was noted between overall toxic leadership and overall nurses silence (*R*-value = -0.769 and *p*-value = 0.001). There was a strong positive significant correlation was found not only between organization performance scale and overall nurses silence (*R*-value = 0.524 and *p*-value = 0.001) but also with all subscales of employee silence scale which are acquiescent silence (*R*-value = 0.527 and *p*-value = 0.001), quiescent silence (*R*-value = 0.395 and *p*-value = 0.001), prosocial silence (*R*-value = 0.433 and *p*-value = 0.001), and opportunistic silence (*R*-value = 0.329 and *p*-value = 0.001). Table 4 revealed a strong, negative significant relation between toxic leadership and nurses silence (*r* = 0.415, *p*-value < 0.001) and organizational performance (*r* = 0.578, *p*-value < 0.001). In addition, Fig. 2 depicted the path analysis model created using SPSS-AMOS, which clarifies the structural equation modeling’s standardized regression weights (Model X2 = 621.4; *p*-value < 0.001); model fit parameters (CFI = 0.83; GFI = 0.85; RMSEA = 0.1918). Toxic Leadership predicted 65% of the variation in nurses’ silence and 87% of the variation in organizational performance. Furthermore, nurses’ silence as a moderating factor accounted for 73% of the variance in organizational performance. All observed variables in the studied model were highly significant at p-value < 0.001, and the study variables had strong estimates.

**Table 3:** Correlation Matrix Between the Study Variables (*n* = 750)

	**Toxic Leadership**					**Organization Performance**	**Nurse Silence**				
	**Intemperate behavior**	**Narcissistic behavior**	**Self-promoting behavior**	**Humiliating behavior**	**Overall**		**Acquiescent silence**	**Quiescent silence**	**Prosocial silence**	**Opportunistic silence**	**Overall**
Intemperate behavior											
** r**											
** p**											
Narcissistic behavior											
**r**	0.797^*^										
**p**	< 0.001^*^										
Self-promoting behavior											
**r**	0.972^*^	0.699^*^									
**p**	< 0.001^*^	< 0.001^*^									
Humiliating behavior											
**r**	0.723^*^	0.232^*^	0.694^*^								
**p**	< 0.001^*^	< 0.001^*^	< 0.001^*^								
**Overall Toxic leadership**											
**r**	0.988^*^	0.878^*^	0.934^*^	0.642^*^							
**p**	< 0.001^*^	< 0.001^*^	< 0.001^*^	< 0.001^*^							
**Organization Performance Scale**											
**r**	-0.666^*^	-0.608^*^	-0.582^*^	-0.483^*^	-0.684^*^						
**p**	< 0.001^*^	< 0.001^*^	< 0.001^*^	< 0.001^*^	< 0.001^*^						
Acquiescent silence											
**r**	-0.615^*^	-0.613^*^	-0.707^*^	-0.057	-0.614^*^	0.527^*^					
**p**	< 0.001^*^	< 0.001^*^	< 0.001^*^	0.119	< 0.001^*^	< 0.001^*^					
Quiescent silence											
**r**	-0.492^*^	-0.823^*^	-0.297^*^	-0.135^*^	-0.610^*^	0.395^*^	0.07				
**p**	< 0.001^*^	< 0.001^*^	< 0.001^*^	< 0.001^*^	< 0.001^*^	< 0.001^*^	0.056				
Prosocial silence											
**r**	-0.608^*^	-0.683^*^	-0.414^*^	-0.525^*^	-0.675^*^	0.433^*^	-0.104^*^	0.893^*^			
**p**	< 0.001^*^	< 0.001^*^	< 0.001^*^	< 0.001^*^	< 0.001^*^	< 0.001^*^	0.004	< 0.001^*^			
Opportunistic silence											
**r**	-0.454^*^	-0.487^*^	-0.250^*^	-0.538^*^	-0.509^*^	0.329^*^	-0.330^*^	0.803^*^	0.970^*^		
**p**	< 0.001^*^	< 0.001^*^	< 0.001^*^	< 0.001^*^	< 0.001^*^	< 0.001^*^	< 0.001^*^	< 0.001^*^	< 0.001^*^		
**Overall Nurse Silence**											
**r**	-0.684^*^	-0.845^*^	-0.501^*^	-0.415^*^	-0.769^*^	0.524^*^	0.121^*^	0.957^*^	0.965^*^	0.877^*^	
**p**	< 0.001^*^	< 0.001^*^	< 0.001^*^	< 0.001^*^	< 0.001^*^	< 0.001^*^	0.001	< 0.001^*^	< 0.001^*^	< 0.001^*^	

r: Pearson coefficient

* Statistically significant at *p* ≤ 0.05

**Table 4:** Path analysis of direct and indirect effects of toxic leadership on organizational performance mediated by nurse silence

Variable 1	Variable 2	Standardized regression weights	S.E	C.R	*P*-value
Toxic Leadership^a^	Nurse Silence	-0.65	.09	-7.42	< 0.001^*^
Nurse Silence	Organizational Performance	0.73	.57	8.67	< 0.001^*^
Toxic leadership^b^	Organizational Performance^b^	-0.87	.63	-8.79	< 0.001^*^

Model X2; significance 621.4; .001

Model fit parameters CFI; IFI; RMSEA (.83; .85; .18)

*r*  Pearson correlation, *CFI*  Comparative fit index, *IFI*  Incremental fit index, *RMSEA*  Root Mean Square Error of Approximation

^a^(*r* = .415, *p* < .001)

^b^(*r* = .578, *p* < .001)

To validate the relation between organizational performance and toxic leadership, a regression analysis was performed, with organizational performance serving as the independent variable and toxic leadership serving as the dependent variable (Table 5). Because there was a difference in the dependent variable, demographic factors (age, educational background, years in the profession, and years of experience in the present unit) were put into the regression equation. According to the regression analysis, nurses’ perceptions of their nursing managers’ toxic leadership behavior, nurses’ age, educational background, years in the profession, and years of experience in the current unit could predict organizational performance (*p*-value < 0.01) and explained 0.75% of the variance in organizational performance.

**Table 5:** Hierarchical linear regression analysis (stepwise) showing predictors of the organizational performance

Variables (Predictors)	B	Beta	t	*P*	95% CI	
					**LL**	**UL**
**Age**	-1.456	-0.550	19.757^*^	< 0.001^*^	-1.601	-1.312
**Educational Qualifications**	-6.548	-0.214	5.411^*^	< 0.001^*^	-8.924	-4.172
**Years of Experience in the Current Working Unit**	-21.790	-0.670	12.455^*^	< 0.001^*^	-25.224	-18.355
**Years of Experience in Nursing**	21.236	0.680	17.640^*^	< 0.001^*^	18.873	23.599
**Overall Toxic leadership **	-0.404	-0.330	10.845^*^	< 0.001^*^	-0.477	-0.331
***R***^**2**^ = 0.751,*F* = 449.493^*^,*p* < 0.001^*^						

F,p: f and *p* values for the model

R^2^: Coefficient of determination

B: Unstandardized Coefficients

Beta: Standardized Coefficients

t: t-test of significance

*OR* Odds ratio, *CI* Confidence interval, *LL* Lower limit, *UL* Upper Limit

^*^Statistically significant at *p* ≤ 0.05

To validate the relation between Nurse Silence and Toxic Leadership, a regression analysis was performed, with Nurse Silence serving as the independent variable and Toxic Leadership serving as the dependent variable (Table 6). Because there was a difference in the dependent variable, demographic factors (educational background and years in the profession) were incorporated into the regression equation. The regression study revealed that nurses’ evaluations of their nursing supervisors’ toxic leadership style, educational background, and years in the profession may predict Nurse Silence (*p*-value < 0.01) and explained 0.73% of the variation in nurse silence.

**Table 6:** Hierarchical Linear Regression Analysis (Stepwise) Showing Predictors of Nurse Silence

Variables (Predictors)	B	Beta	t	*P*	95% CI	
					**LL**	**UL**
**Educational Qualifications**	6.334	0.266	14.011^*^	< 0.001^*^	5.446	7.221
**Years of Experience in nursing**	-10.889	-0.448	17.873^*^	< 0.001^*^	-12.085	-9.693
**Overall Toxic leadership **	-0.482	-0.505	20.466^*^	< 0.001^*^	-0.528	-0.436
***R***^**2**^ = 0.743,*F* = 719.003^*^,*p* < 0.001^*^						

F,p: f and *p* values for the model

R^2^: Coefficient of determination

B: Unstandardized Coefficients

Beta: Standardized Coefficients

t: t-test of significance

*OR* Odds ratio, *CI* Confidence interval, *LL* Lower limit, *UL* Upper Limit

^*^ Statistically significant at *p* ≤ 0.05

## Discussion

The current study investigated the relationship between toxic leadership and organizational performance, as well as the function of nurses’ silence in Egypt as a mediator. The findings confirmed that the participating nurses experienced toxic leadership. According to Abou-Ramadan and Eid (2020) [37], more than one-third of nursing staff judged their leaders to have a high and moderate degree of narcissism, as well as unpredictable toxic leadership behaviours. This finding contradicted the findings of Labrague et al., (2021) [38], who found that nurse managers believed their leadership behaviours to be “non-toxic”. For example, in research involving 1127 clinical nurses in China, nurse supervisors were rated as non-abusive leaders by staff nurses [39]. In the current investigation, the overall score for nurse silence was moderate. Indeed, this might be explained by the presence of toxic leadership at a high level. This finding was consistent with the findings of a research done at Jordanian capital health settings, which found that staff experience a moderate to high level of perceived general organizational silence [40]. Another survey done in the same university hospital in Egypt [33] found that the average perceived overall organizational silence level is moderate. The most unexpected conclusion in the current study was that nurses exhibit excellent levels of performance while working in a hazardous atmosphere and practicing silence. Indeed, Tepper (2007) [41] and Xu et al., (2015) [42] explained that negative leadership behaviors cause stress and emotional exhaustion among employees, and as a result, employees try to conserve their knowledge resources and physical resources in order to cope with the adverse climate created by the leaders, and instead of directing their efforts toward the achievement of organizational goals and performance, they waste their strenuous efforts. As a result, it might be claimed that they are trying hard to reduce the influence of their toxic leaders.

The study discovered a considerable negative correlation between toxic leadership and organizational performance. This finding was congruent with the findings of Kiliç and Günsel (2019) [43], who said that toxic leadership may lead to a drop in workplace performance, productivity, and output, as well as significant negative effects on workers. Khan et al., (2021) [44] and Saqib & Arif, (2017) [45] mentioned the same unfavourable associations. In contrast to this viewpoint, Ferris et al., (2007) [46] observed that dysfunctional CEOs can have good results for firms in the short run. The new findings may help nursing executives understand how to help and support mistreated personnel. Furthermore, there are demands to prevent toxic leadership behaviours, which may have a detrimental influence on nurses’ organizational performance.

The researchers discovered a high, negative, and significant correlation between overall toxic leadership and overall nurse silence in the current study. Contrary to popular belief, Saqib and Arif (2017) [45] demonstrated that toxic leadership behaviours have a considerable positive influence on nurse silence. Furthermore, Xu et al., (2015) [42] discovered a favourable relationship between toxic leadership and nurse silence. As a result, it can be concluded that toxic leadership not only impacts an individual’s job but also increases the quiet of nurses. The regression models used in the study demonstrated that toxic leadership has an influence on organizational performance as well as on nurses’ silence. As a result, the nurse’s silence served as a mediator.

## Strengths and limitations

The findings of this study significantly added to existing research on toxic leadership, organizational performance, and nurses’ silence. The study, however, should be interpreted in light of its limitations. The participants were drawn from a specific setting for convenience, so the generalizability of the results is limited. Furthermore, because the current results were based on self-reported data, they were vulnerable to response bias and subjectivity. Furthermore, this study only showed correlations between study variables; no causal relationship can be established. In the future, longitudinal, experimental, and multi-site research may help to address these limitations. The current study had several advantages; as the cross-sectional method allowed for the simultaneous measurement of multiple variables in a population sample, resulting in more reliable data that was less susceptible to the potential biases of case series and case reports. A longer follow-up could had aided the investigation. Finally, no claim was made about the relationship between the variables in the study, its purpose was to look into the relationship between variables. Future research should focus on specific strategies or treatments for dealing with nurses’ silence and toxic leadership. Future research can also test job satisfaction, organizational commitment, and work engagement. Furthermore, researchers and practitioners will be more concerned with determining why leaders are toxic and advising on how to control and manage these behaviours.

## Implications of the study

Toxic nursing leadership behaviours that endanger patient safety must be addressed organizationally. First, during their transition period, nurse managers and nurses, particularly those with less experience, may benefit from structured mentorship, coaching, and feedback from experienced nurse managers. While standalone nurse and nurse manager transition programs (for example, orientation programs, preceptorship and mentorship initiatives) can assist new nurses and nurse managers in making the transition to practice, a multifaceted nurse manager transition program may be required because it captures various essential elements of the transition experience [47]. When evaluating applicants for nurse management positions, use leadership assessment scales to assess the need for emotional intelligence and leadership qualities. Furthermore, frequent evaluation of nurse managers’ performance, whether through bottom-up performance assessments or the use of the 360-degree feedback method, may highlight both good and bad leadership techniques. A zero-tolerance policy for toxic and other similar behaviours, as well as a policy describing appropriate workplace behaviours, may help to reduce the spread of harmful workplace behaviours [48]. Nurse managers should create a safe and stress-free environment for healthcare workers to express their ideas without fear of being criticized by colleagues and superiors, and they should encourage their employees to express their opinions by providing proper mechanisms for free expression and constructive criticism. The negative attitude of senior managers toward employees’ comments and feedback further limits opportunities for communication and exchange between senior managers and employees, intensifying silent behaviours on their behalf [49].

## Conclusion

This study was regarded as the research to evaluate toxic leadership and organizational silence and their impact on organizational performance among Egyptian nurses. According to the findings of this study, toxic leadership had a highly statistically significant negative relationship with organizational performance, as well as a highly statistically significant negative relationship with nurses’ silence. Nurses in various hospital units should receive targeted training to improve their understanding of toxic leadership and nurse silence predictors. Furthermore, the study’s findings emphasized on the importance of creating a work environment that encourages and promotes open communication, as well as eliminating toxic leadership behaviours from the organizational culture among nurses. It is critical that organizational leaders address and initiate programs to enable nurse involvement and reduce nurse silence, as well as encourage a culture of reporting and collaborative communication among nurses and their leaders, as well as reduce the negative impact of silence on their leaders’ negative and toxic behaviours, and train them on how to deal with various toxic behaviours that may affect their performance. Furthermore, the findings confirmed the importance of managerial caring in promoting nurse communication, collaboration, and performance, as well as building a motivating nursing workforce.

## Data Availability

The datasets generated and/or analysed during the current study are not publicly available due to data privacy but are available from the corresponding author on reasonable request.
